# Restoration of soils contaminated with PAHs by the mixture of zeolite composites mixed with exogenous organic matter and mineral salts

**DOI:** 10.1038/s41598-023-41429-2

**Published:** 2023-08-30

**Authors:** Justyna Szerement, Adam Kowalski, Jakub Mokrzycki, Lidia Marcińska-Mazur, Monika Mierzwa-Hersztek

**Affiliations:** 1 Department of Radiochemistry and Environmental Chemistry, Maria Curie-Sklodowska University, 3 Maria Curie-Skłodowska Square, 20-031 Lublin, Poland; 2 Department of Environmental Analysis, Geological Mapping and Economic Geology, AGH University of Science and Technology, Mickiewicza 30 Av., 30-059 Kraków, Poland; 3 Department of Coal Chemistry and Environmental Sciences, Faculty of Energy and Fuels, AGH University of Science and Technology, Mickiewicza 30 Av., 30-059 Kraków, Poland; 4 Department of Mineralogy, Petrography and Geochemistry, AGH University of Science and Technology, Mickiewicza 30 Av., 30-059 Kraków, Poland; 5 Department of Agricultural and Environmental Chemistry, University of Agriculture in Krakow, Mickiewicza 21 Av., 31-120 Kraków, Poland

**Keywords:** Environmental sciences, Environmental monitoring, Pollution remediation, Risk factors

## Abstract

The major cause of soil degradation (contamination, erosion, compaction) is closely linked to agriculture, i.e., unsustainable agriculture practices, which are reflected in the depletion of the soil organic carbon pool, loss in soil biodiversity, and reduction of C sink capacity in soils. Therefore, the agricultural practice of applying carbon-rich materials into the soil is an attractive solution for climate change mitigation and soil ecosystem sustainability. The paper aimed to evaluate the effectiveness of the addition of organic-mineral mixtures to the mineral salts (NPK), including the exogenous organic matter (lignite) mixed with zeolite-carbon (NaX-C) or zeolite-vermiculite (NaX-Ver) composites in the restoration of soils contaminated with PAHs. The addition of zeolite composites to fertilizer resulted in a significant reduction in soil PAH levels and a corresponding reduction in plant tissue content, without compromising yields, compared to the control and separate application of NPK. A Significant correlation between PAHs and pH_H2O_, pH_KCl_, EC and dehydrogenase activity (DhA) was found in soils. The addition of zeolite composites with lignite significantly reduced the content of PAHs in straws, especially following the application of NaX-C. However, in the case of grains, the highest percentage reduction in comparison to NPK was observed for the highest dose of NaX-Ver.

## Introduction

It is estimated that a third of the world's soil is moderately to highly degraded^1^. Of all the types of soil degradation, chemical soil degradation (caused by the presence of heavy metals, organic contaminants etc.) is recognised as one of the most prevalent worldwide^2^, and its growing number is closely linked to unsustainable agriculture practices reflected in the depletion of the soil organic carbon (SOC) pool, loss in soil biodiversity, and decrease in soil fertility and elemental imbalance^3^.

Polycyclic aromatic hydrocarbons (PAHs) are a large group of persistent, hydrophobic organic compounds containing two or more aromatic rings^4^. They can be classified according to their benzene ring numbers into two groups: 2–3-rings for low molecular weight (LMW) and 4-,5-, and 6-rings for high molecular weight (HMW)^5^. Extensive accumulation of PAHs in soils leads to serious agricultural and environmental issues around the world^6^. PAHs in crops may directly exert adverse impacts on the quality and safety of agricultural products and result in potential risks to human health^7^. These pollutants are highly toxic to soil microorganisms^8^. Microorganisms and soil enzymes can decompose benzene ring chains in PAHs^9^. For example, the dehydrogenase activity (DhA) can be used to evaluate the degradation performance^10^. It was also evidenced that microorganisms can take part in the regulation of SOC decomposition and storage, thus playing an important role in organic matter turnover and nutrient cycling^11^. Highly contaminated soils are usually poor in soil organic matter (SOM) and microbial activity. SOM, often estimated and expressed as SOC^12^, acts as a large carbon sink, and carbon farming as one of the land management practices that reduce greenhouse gas emissions and increase the sequestration and storage of carbon in soils and vegetation^13,14^. Additionally, it is one of the most important components of soil, essential for sustaining high levels of food production^15^. Unfortunately, the current rate of carbon loss due to*.* unsustainable agriculture practices corresponds to 1.5 (1.0/1.8) GT carbon per year^16^.

To restore the appropriate agricultural suitability of soils, degraded soil must be remediated and conserved using simple and cost-effective approaches^17^. These approaches should also include sustainable agriculture, which recommends reducing the quantity of chemical fertilizers used in their sector without compromising yields and promoting techniques that generate co-benefits in terms of adaptation, mitigation, and increased food production^18^.

One of the promising approaches recommended to offset the loss of SOM content and crop productivity is the application of exogenous organic materials containing organic carbon such as lignite and nutrients^19^. As an organic additive, lignite is rich in humic and fulvic acids ^20^. It was also proved that zeolite (natural and synthetic) as an additive to fertiliser can improve the physical, chemical, and biological properties of soil^21–24^. Previous studies have shown that adding exogenous organic matter mixed with zeolite-carbon composite (NaX-C) and zeolite-vermiculite composite (NaX-Ver) can stimulate soil microbial activity^25–27^ and also have a positive effect on root morphological parameters^28^. However, information about the application of these additives to the soil contaminated with PAHs is limited. It was evidenced that NaX-C and NaX-Ver mixed with leonardite reduced the content of PAHs in soil and tissues of maize^29^; however, there is no information on the effect of zeolite composites combined with other exogenous organic matter, like lignite, on the content of PAHs in soils and their plant accumulation.

The aim of the paper was the evaluate the effectiveness of the addition of organic-mineral mixtures to the mineral salts (NPK), including the lignite mixed with NaX-C or NaX-Ver composites on changes in content of total organic carbon (TOC), DhA, pH, EC, total N (TN), level of the degradation of PAHs, and uptake and distribution of PAHs in maize plant cultivated on PAHs-contaminated soil.

## Results

### Soil properties

According to the World Base for Soil Resources (WRB), the soil was Eutric Cambisol (CM-eu), with 85%, 12% and 3% of sand, silt and clay (loamy sand), respectively (Table 1). The soil was acidic, with a pH value of 5.24. The content of TOC was 6.34 g kg^−1^. Based on the European classification system of soil contamination^30^, the sum of 16 analysed PAHs (Σ16 PAHs) in soils represents heavy pollution. The content of Cd can be defined as a I degree of soil contamination, and on the basis of Zn and Pb, as a II degree of soil contamination^31^. The value of the pH significantly decreased in fertilized objects in comparison to the control; however, no significant differences (p > 0.05) were observed between types of fertilization (Table 2). The EC value was the lowest for C9L6 (305.25 ± 60.52 µS cm^−3^). Generally, in all soils with fertilization, the DhA was lower compared to the control (0.85 μg TPF g^−1^ h^−1^), except for C9L6. There were no significant differences in the TOC between control and fertilized objects, except C3L3. Additionally, there was a positive correlation between DhA and TOC (0.60, p < 0.05). The BC varied in soils from 5.49 ± 0.39 for V3L3 to 6.50 ± 0.42 for C3L3. The TN did not vary between variants. The ratio TOC:TN was higher for all variants with fertilization in comparison to the control, with the highest value for V3L3. Pearson’s correlation coefficients of the pH, EC, BC, TOC, DhA and 2, 3, 4, 5, and 6-rings PAHs are summarised in Table 3.

**Table 1:** Selected properties of the soil used in the experiment.

Soil parameter	Value	Unit
Sand	85	%
Silt	12	%
Clay	3	%
pH_H2O_	5.24	–
pH_KCl_	5.03	–
EC	273	μS cm^−1^
BC	0.65	%
TOC	6.34	g kg^−1^ d.m
N_total_	0.40	
C_total_	5.74	
S_total_	0.118	
Pb_total_	188	mg kg^−1^ d.m
Cd_total_	1.15	
Zn_total_	267	
PAHs	1.3

**Table 2:** Selected soil characteristics after the experiment.

Variants	C	NPK	C3L3	C9L6	V3L3	V9L6
Properties						
pH_H2O_	5.99 ± 0.06^a^	5.62 ± 0.17^b^	5.57 ± 0.29^b^	5.58 ± 0.03^b^	5.49 ± 0.39^b^	5.81 ± 0.61^b^
pH_KCl_	5.26 ± 0.07^a^	4.81 ± 0.08^b^	4.87 ± 0.08^b^	4.89 ± 0.05^b^	4.88 ± 0.01^b^	4.94 ± 0.13^b^
EC, μS cm^−1^	398.25 ± 45.41^cd^	416.67 ± 55.44^bcd^	531.33 ± 82.68^ab^	305.25 ± 60.52^d^	561.00 ± 35.36^a^	476.00 ± 41.82^abc^
DhA, μg TPF g^−1^ h^−1^	0.85 ± 0.14^a^	0.37 ± 0.09^b^	0.21 ± 0.04^b^	0.69 ± 0.07^a^	0.35 ± 0.02^b^	0.29 ± 0.09^b^
TOC, g kg^−1^ d.m	6.08 ± 0.35^a^	5.61 ± 0.36^a^	5.18 ± 0.20^b^	5.97 ± 0.20^a^	5.96 ± 0.29^a^	6.21 ± 0.31^a^
BC, g kg^−1^	6.39 ± 0.22^ab^	6.37 ± 0.35^ab^	6.50 ± 0.42^a^	6.33 ± 0.45^ab^	5.49 ± 0.39^b^	5.81 ± 0.61^ab^
TN, g kg^−1^	0.5 ± 0.05	0.42 ± 0.09	0.42 ± 0.01	0.42 ± 0.08	0.40 ± 0.10	0.47 ± 0.06
TOC:TN	12.3	17.9	16.9	17.1	19.4	16.8
Cd_total,_ mg kg^−1^ d.m	1.06 ± 0.08^ab^	0.79 ± 0.08^c^	0.89 ± 0.14^c^	0.91 ± 0.03^abc^	0.89 ± 0.01^bc^	1.07 ± 0.04^a^
Zn_total,_ mg kg^−1^ d.m	241.53 ± 16.1^ab^	174.88 ± 23.7^c^	220.30 ± 18.35^b^	213.16 ± 5.59^b^	210.43 ± 6.90^b^	255.33 ± 12.45^a^
Pb_total,_ mg kg^−1^ d.m	172.69 ± 10.28^a^	154.39 ± 5.94^bc^	165.96 ± 8.60^ab^	165.30 ± 8.67^ab^	144.10 ± 1.44^c^	179.53 ± 8.04^a^

Data represent means ± SD of four replications. Different letters in the rows indicate a significant difference (p < 0.05) according to the HSD Tukey test for α = 0.05.

**Table 3:** Pearson’s correlation coefficients between PAHs content and soil characteristics.

Soil parameter	2-rings	3-rings	4-rings	5-rings	6-rings
pH_H2O_	0.73*	0.58*	0.53*	0.59*	0.61*
pH_KCl_	0.61*	0.49*	0.55*	0.68*	0.75*
EC	− 0.45*	− 0.62*	− 0.66*	− 0.57*	− 0.57*
DhA	0.65*	0.60*	0.71*	0.74*	0.78*
TOC	0.09	0.06	0.34	0.43*	0.42*
TN	− 0.46*	− 0.67*	− 0.64*	− 045*	− 0.52*
BC	0.32	0.27	0.15	0.15	0.17
Cd_total_	0.19	0.06	0.31	0.40	0.51*
Zn_total_	0.11	− 0.07	0.24	0.34	0.44*
Pb_total_	0.47*	0.38	0.45*	0.42*	0.53*

Asterisks indicate a significant difference (p < 0.05).

### Content of PAHs in soils

**Figure 1:**
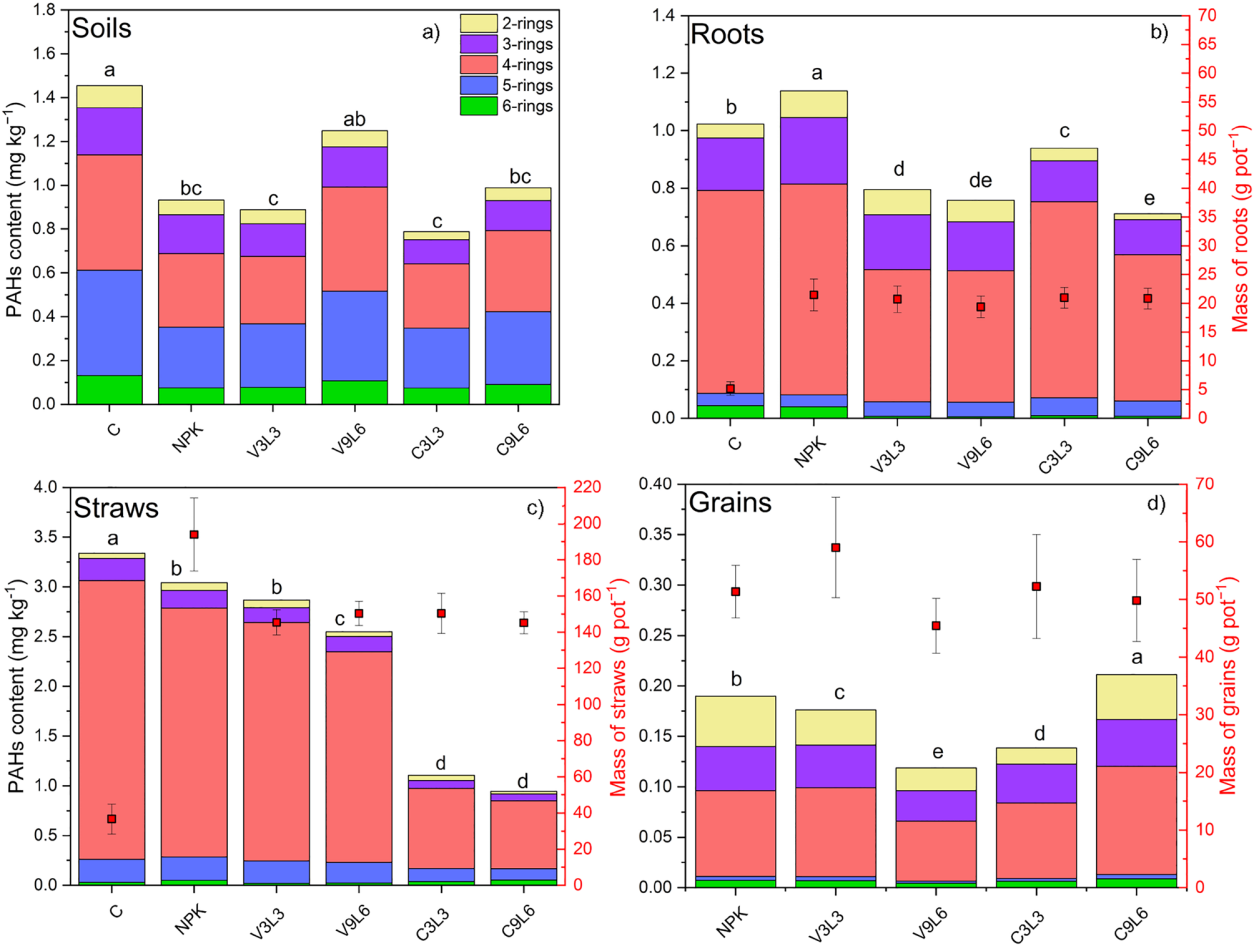
Concentration of 2, 3, 4, 5, 6-rings PAHs in soils (**a**), roots (**b**), straws (**c**) and grains (**d**) of maize (in mg kg^−1^) and mass of roots, straws, and grains (in g pot^−1^, black square with SD). Different letters on bars indicate a significant difference in Σ16 PAHs (p < 0.05) according to the HSD Tukey test for α = 0.05.

The concentration of 2-, 3-, 4-, 5-, and 6-rings PAHs in soils is presented in Fig. 1a. The content of individual PAHs is also shown in Table S1. All soil samples contained all rings of PAHs, with HMW PAHs contributing the highest percentage of total PAHs (ranging from 73.7 to 84.0%). The contribution of HMW and LMW differed among objects with zeolite composites amendments, and the lowest ratio between HMW and LMW was observed for the application of Ver3L3 (3.18). In fertilized soils, the Σ16 PAHs were significantly lower (p > 0.05) in comparison to the control. (1.45 ± 0.17) and ranged from 0.79 ± 0.05 mg kg^−1^ for C3L3 to 1.24 ± 0.29 mg kg^−1^ for V9L6.

### Content of PAHs in plant tissues

The concentration of 2-, 3-, 4-, 5-, and 6-rings PAHs in roots (Fig. 1b), straws (Fig. 1c) and grains (Fig. 1d) is presented in Fig. 1 and Table S1. The content of Σ16 PAHs changed in the following order: straws > roots > grains. The 4-ringed PAHs were the most predominant group, and their highest content was observed in the straws (from about 72.2% in the C9L6 to 90.0% in the control). The application of both zeolite composites mixed with lignite significantly reduced the Σ16 PAHs in maize roots (from 8.21 to 30.5% and from 17.5 to 37.5% in comparison to control and NPK, respectively) with simultaneously no reduction in mass of roots. The application of both zeolite composites mixed with lignite significantly reduced the content of 6-rings of PAHs in roots by about 78.84% for C3L3 to 87.18% for V9L6 compared to NPK. In straws, the highest reduction of 4-, 5-, and 6-rings PAHs was observed for application of NaX-C, especially when applied at a higher dose (69.26%, 66.13%, 59.44%). For grains, the lowest content of Σ16 PAHs was observed for V9L6 (0.12 ± 0.02 mg kg^−1^). There was no grain yield in the control variant.

**Figure 2:**
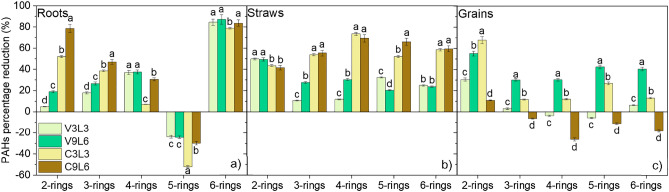
Percentage reduction of the content of 2, 3, 4, 5, and 6-rings PAHs in roots (**a**), straws (**b**), and grains (**c**) of maize after application of zeolite composites mixed with lignite against the PAHs content in maize tissues for the application of the conventional fertilization NPK.

Figure 2 shows the reduction of content of the 2-, 3-, 4-, 5-, and 6-rings PAHs in roots, straws, and grains compared to results obtained after NPK fertilization. In roots, the highest reduction was observed for 6-rings PAHs (Fig. 2a) (from 78.84% for C3L3 to 83.70% for V3L3), whereas a significant increase of 5-rings PAHs was observed (up to 52.17% for C3L3). In the case of straws, the highest reduction HMW was observed for NaX-C (more than 52%). Contrasting results were obtained for grains, where the highest decrease of HMW was observed for NaX-Ver, but only for the higher dose of mineral-organic mixture. The application of V9L6 reduced the level of 4-, 5, and 6-rings PAHs by 30.33%, 42.44%, and 40.51%, respectively, whereas the application of C9L6 increased the level of these PAHs by 26.26%, 11,09%, and 18.24%. The lowest content of BaP_eq_ and BaP with the most carcinogenic properties in grains was observed in V9L6 (0.89 ± 0.03 and 0.82 ± 0.03) (Table 4.).

**Table 4:** Content of BaP and value of the BaP_eq_ in maize grains.

	BaP (μg kg^−1^)	BaP_eq_ ( μg-BaP kg^−1^)
NPK	1.45 ± 0.05^a^	1.59 ± 0.05^a^
V3L3	0.89 ± 0.03^c^	1.08 ± 0.04^c^
V9L6	0.82 ± 0.03^c^	0.89 ± 0.03^d^
C3L3	0.86 ± 0.03^c^	0.97 ± 0.03^d^
C9L6	1.36 ± 0.04^b^	1.49 ± 0.05^b^

Data represents means ± SD of four replications. Different letters in the columns indicate a significant difference (p < 0.05) according to the HSD Tukey test for α = 0.05.

### Bioaccumulation factors (BFs)

The mobility of PAHs in soil and their availability in plants were calculated as bioaccumulation factors (BFs). The bioaccumulation factors of 2, 3, 4, 5, and 6-rings PAHs for maize plant tissues are presented in Table 5. The highest value of root bioconcentration factors (RBFs) and straw bioconcentration factors (SBFs) for 6-rings PAHs was observed for NPK. The lowest value of grain bioconcentration factors (GBFs) for 2- (0.40 ± 0.08), 3- (0.19 ± 0.04), 4- (0.16 ± 0.01), 5- (0.007 ± 0.001), and 6-rings PAHs 0.049 ± 0.006) was observed after the application of V9L6.

**Table 5:** Bioaccumulation factors (BFs) of 2, 3, 4, 5, and 6-rings PAHs for roots, straws and grains of maize.

Object	2-rings	3-rings	4-rings	5-rings	6-rings
RBFs					
NPK	1.43 ± 0.31^a^	1.34 ± 0.24^ab^	2.20 ± 0.21^ab^	0.15 ± 0.01^b^	0.541 ± 0.041^a^
V3L3	1.76 ± 0.10^a^	1.57 ± 0.25^a^	1.45 ± 0.10^c^	0.17 ± 0.01^b^	0.074 ± 0.064^bc^
V9L6	1.32 ± 0.16^a^	1.07 ± 0.24^b^	1.23 ± 0.11^c^	0.16 ± 0.02^b^	0.057 ± 0.007^c^
C3L3	0.81 ± 0.04^b^	1.18 ± 0.21^ab^	2.28 ± 0.09^a^	0.21 ± 0.01^a^	0.106 ± 0.007^b^
C9L6	0.50 ± 0.22^b^	1.18 ± 0.14^ab^	1.94 ± 0.03^b^	0.21 ± 0.01^a^	0.090 ± 0.001^bc^
SBFs					
NPK	0.78 ± 0.15^b^	1.36 ± 0.23^ab^	7.53 ± 0.78^a^	0.64 ± 0.07^a^	1.029 ± 0.091^a^
V3L3	0.39 ± 0.04^c^	1.85 ± 0.30^a^	7.55 ± 0.52^a^	0.49 ± 0.04^b^	0.895 ± 0.077^a^
V9L6	0.40 ± 0.08^c^	1.31 ± 0.30^b^	5.70 ± 0.56^b^	0.48 ± 0.08^b^	0.504 ± 0.069^bc^
C3L3	0.69 ± 0.06^bc^	1.10 ± 0.21^b^	2.69 ± 0.12^c^	0.27 ± 0.01^c^	0.638 ± 0.039^b^
C9L6	1.35 ± 0.33^a^	1.10 ± 0.14^b^	2.60 ± 0.08^c^	0.26 ± 0.02^c^	0.393 ± 0.007^c^
GBFs					
NPK	0.77 ± 0.15^b^	0.25 ± 0.05^bc^	0.26 ± 0.03^b^	0.014 ± 0.001^b^	0.100 ± 0.009^b^
V3L3	0.69 ± 0.08^bc^	0.35 ± 0.06a^b^	0.28 ± 0.02^b^	0.013 ± 0.001^b^	0.084 ± 0.007^c^
V9L6	0.40 ± 0.08^ cd^	0.19 ± 0.04^c^	0.16 ± 0.01^c^	0.007 ± 0.001^d^	0.049 ± 0.006^d^
C3L3	0.30 ± 0.02^d^	0.32 ± 0.06^b^	0.25 ± 0.01^b^	0.009 ± 0.001^c^	0.081 ± 0.005^c^
C9L6	1.13 ± 0.27^a^	0.45 ± 0.05^a^	0.41 ± 0.01^a^	0.016 ± 0.001^a^	0.121 ± 0.001^a^

Data represents means ± SD of four replications. Different letters in the columns indicate a significant difference (p < 0.05) according to the HSD Tukey test for α = 0.05.

## Discussion

The degradation of PAHs in soils is a complex process and depends on many factors, such as soil type, temperature, pH, and soil organic matter content^32^. In the present study, the application of both zeolite composites mixed with lignite generally decreased the level of all the rings-PAHs in soils in comparison to the control. However, the ratio HMW:LMW for these soils increased, suggesting that LMW PAHs were more easily degraded than HMW PAHs. The HMW PAHs are more resistant to degradation by bacterial or litter-decomposing fungi than LMW PAHs^31,32^. Additionally, the HMW PAHs represented the majority of the total PAHs in tested soils (from 73.7 to 84.0%), which is in line with the results obtained by Ukalska-Jaruga et al.^33^. In the presented study, a significant positive correlation between 5-, and 6-rings PAHs and TOC was found to be consistent with the results shown by Duan et al.^34^. The PAHs with a higher number of rings have a higher octanol–water partitioning coefficient (logK_ow_) compared to PAHs with a lower number of rings, resulting in a lower tendency to dissolve in water and more tendency to adsorb onto the organic matter^35^.

There were no statistical differences between the content of TN in tested soils. For better characterisation of the quality of SOM, the TOC/TN ratio was calculated (Table 2). The potential decomposition rate of organic C and potentially mineralizable N was higher in soils with high organic matter input. The TOC/TN ratio was higher in soils with all fertilization and showed diversified values from 12.3 for the control to 17.1 for V3L3, which indicates a varying degree of organic matter decomposition in tested soils. It was also found that the content of the TN in soils had a significant negative correlation with the content of the 2-, 3-, 4-, 5-, and 6-rings PAHs, which is opposite to results reported by Han et al.^36^, where TN had significant positive correlation with HMW PAHs.

The addition of mineral-organic mixtures, rich in organic carbon, did not change the level of TOC in tested soils except for C3L3, where the level of the TOC decreased statistically. Generally, the TOC levels change slowly and are insensitive to vegetation restoration over short periods in natural conditions^37^. However, the exogenous addition of SOM to the soil can stimulate soil microorganisms, which decompose benzene ring chains in PAHs ^9^. Microorganisms rapidly consume nutrients released after the first stages of hydrocarbon degradation^38^; hence, at the medium and long-term phases, when nutrients release is reduced considerably, carbon and nutrient concentrations in hydrocarbon-polluted soils may decrease significantly. The DhA in soils are usually the most sensitive indicator of environmental changes, and their activities are always affected by soil conditions through shifting the synthesis and structure of local microorganisms^39^. It was also evidenced that zeolites, due to their high porosity and well-developed specific surface area, can improve the soil’s microbial activity^26,27, 40^. The DhA in amended soils was lower compared to the control, except for C9L6. Additionally, there were no statistical differences between fertilization. The lower DhA in soils after the maize harvest might have resulted from a lower availability of soluble organic carbon^41^. Additionally, the pot experiment took nearly 5 months, and the highest DhA was observed after 3 months in the incubation experiment (data not shown), particularly for the application of both zeolite composites mixed with lignite. It was also evidenced that NaX-C and NaX-Ver combined with leonardite or lignite stimulated the microbial activity of unpolluted soils^26,42, 43^.

Soil amendments (organic and inorganic) may change the soil pH, which can have an indirect effect on the bioavailability of PAHs and control the soil's biology and biological processes^44^. A significant positive correlation between soil pH_H2O_/pH_KCl_ and PAHs suggested that the soil’s pH was a key factor in the soil PAHs levels. The statistical negative correlation of all rings PAHs with EC suggest that this factor also indirectly affects the degradation of PAHs in soils^45^. The same results were observed for the application of zeolite composites mixed with leonardite^29^.

In the presented study, the 2-, 3-, 4-, 5-, and 6-rings PAHs were significantly correlated with Pb, and for 6-rings PAHs with Zn and Cd. Heavy metals can make cation-π-bonds with PAHs^46^. Results showed by Oste et al.^47^ indicated that the alkaline pH of the synthetic zeolites caused an increase in the content of the dissolved organic matter, affecting improved sorption of HMs by the solid phase.

Generally, applying both zeolite composites combined with lignite reduced the level of PAHs significantly in roots, straws and grains (except C9L6) compared to NPK applied separately. The highest values of the RBFs, SBFs, and GBFs were obtained for 4-rings PAHs, with pyrene being the most dominant. Pyrene was mainly accumulated in straws of maize, in contrast to wheat cultivated in a hydroponic system, where pyrene was accumulated primarily in roots^48^. The lower SBFs for 4-, 5- and 6- rings PAHs for both zeolite composites mixed with lignite in comparison to NPK (more than two times lower in the case of zeolite-carbon composites) suggested that less HMW PAHs were transported from soil to straws of maize which is in line with the results showed by Tao et al.^49^ for wheat roots, and Szerement et al.^29^ for maize. The addition of both zeolite composites combined with lignite changed the properties of the soil, which could have affected the composition of root exudates (low-molecular-weight organic acids)^50^. Thus, the lower uptake of PAHs from soil can also be connected to mechanisms between microorganisms and plant abilities to stimulate microbial PAH degradation by root exudates. However, the role of root exudates in the degradation of PAHs is still not fully understood^51^.

Grains exhibited the lowest concentration of PAHs compared to straws and roots. Similarly to the results obtained for zeolite composites mixed with leonardite (− 0.81, p < 0,05)^29^, a significant negative correlation was found between the content of PAHs in soil and grains (− 0.58, p < 0.05). Thus, it was evidence that PAHs in grains were mainly accumulated from the air; however, depending on applied zeolite composites, the level of PAHs in grains varied. It is further confirmation that the type and amount of zeolite composites play a significant role in altering soil properties and the maize plant's ability to accumulate PAHs. According to US-EPA, BaP is recognized as the most dangerous pollutant due to its high carcinogenic and mutagenic character^52^. The application of both zeolite composites mixed with lignite significantly reduced the level of BaP and BaP_eq_ in maize grains compared to NPK. In the case of BaP, the best results were obtained for the lower dose of NaX-C (C3L3) and both doses of NaX-Ver (V3L3, V9L6), i.e., reduction of BaP by approximately 40.69%, 43.51, and 40.65, respectively in comparison to NPK. These trends are in line with results obtained for the application of the zeolite composites mixed with leonardite^29^. However, applying the lower dose of zeolite-carbon composite mixed with leonardite has about 33% better effect on the reduction of BaP than lignite used in the current experiment.

Based on the obtained results, it can be concluded that adding a mixture of zeolite composites mixed with lignite and mineral salts can be an innovative and effective way to restore soil contaminated with PAHs and limit their uptake by plants. Another advantage of their application is that they can reduce the use of mineral fertilizers, which is one of the main strategies of sustainable agriculture. However, the main disadvantage is their high salinity and low structure stability at the lower pHs. Thus long-term studies are needed to better understand their impact on soil changes^53^.

## Materials and methods

### Chemical reagents

Ammonium nitrate (NH_4_NO_3_), calcium dihydrogenphosphate monohydrate (Ca(H_2_PO_4_)_2_ H_2_O); potassium chloride (KCl), sodium sulfate (Na_2_SO_4_), acetone, anhydrous sodium sulfate (Na_2_SO_4_), triphenyltetrazolium chloride (TTC), acetone were purchased from S.A. POCH, Poland. N-Hexane (HEX), acetone (ACE), dichloromethane (DCM), and methanol (MeOH) with purity > 99.9% used for chromatographic analyses were purchased from Chemsolute. Standard of 16 PAHs in a 2000 μg ml^−1^ mixture solution in DCM (CRM47930), deuterated PAHs internal standard solutions (phenanthrene-d10 at concentration 2000 μg ml^−1^ in DCM) were obtained from Sigma-Aldrich. Standard working solutions of PAHs mixture, internal standard mixture and phenanthrene-d10 were diluted properly with dichloromethane (DCM) and prepared freshly before the analysis.

### Characteristics of soil samples

The soil (upper soil layer of 0–30 cm) used in the experiment was sampled from an agriculture field located near a coniferous forest in South Malopolska, Poland (50°05′35.9" N 19°39′52.9" E). Next, the soil was air-dried, sieved (2 mm) and manually homogenized.

### Pot experiment and sampling

A pot experiment was carried out in 2020 in the vegetation hall with a transparent glass roof to prevent precipitation but ensure natural light and ventilation (Faculty of Agriculture and Economics of the University of Agriculture in Krakow-Mydlniki). PVC pots (25 cm height, 22 cm diameter) were filled with 9 kg of air-dried soil. During the experiment, the soil water content was maintained at the maximum water holding capacity using a Rain Bird sprinkler system (Rain Bird Inc., Tucson, USA) equipped with a soil moisture sensor. Soil moisture was controlled using a portable probe with an ECH2O EC5 sensor (Decagon Devices, Pullman, Washington, USA).

The soils were mixed with six different treatments in four independent replications. The reference variant was a control object without fertilization: **C**—control soil (without fertilization), and **NPK,** 100% NPK—soil with an addition of mineral salts: N—NH_4_NO_3_; P—Ca(H_2_PO_4_)_2_ H_2_O; K—KCl in doses of 0.20 g N kg^−1^, 0.10 g P kg^−1^, and 0.25 g K kg^−1^ for N, P, K, respectively. The objects with the addition of the mineral-organic mixtures (zeolite composites mixed with lignite) were as follows: **V3L3**—soil with a mixture of the 3% of zeolite-vermiculite composite, 3% of lignite, and 94% NPK; **V9L6**—soil with a mixture of the 9% of zeolite-vermiculite composite, 6% of lignite, and 85% NPK; **C3L3**—soil with a mixture of the 3% of zeolite-carbon composite, 3% of lignite, and 94% NPK; **C9L6**—soil with a mixture of the 9% of zeolite-carbon composite, 6% of lignite, and 85% NPK. Each pot had 15 seeds of maize variety of Kosynier (provided by Centrala Nasienna Krakow) sown and then left to 5 seedlings after germination. Due to visible symptoms of N deficiency during vegetation, the supplementary NH_4_NO_3_ at a dose of 0.02 g N kg^−1^ dry mass (d.m.) of soil was applied. Soil moisture during plant vegetation was maintained at 40% to 60% of the maximum water capacity of the soil (depending on the development phase of the plant).

In the fully ripe stage of the maize—on day 126 post-planting, the plants were harvested, and the roots were collected from the soil and washed with distilled water to remove soil and other debris. The dry mass was calculated after drying straws (stalks + leaves), roots and cobs at 65 °C for 24 h. Soil samples were collected from each pot, sieved and stored for further analyses at 4 °C and 25 °C for biological and physicochemical analyses, respectively.

The temperature and humidity were recorded from March to September outside the vegetation hall (Table 6).

**Table 6:** Average monthly temperature and air humidity.

Month	Temperature (^o^C)	Air humidity (%)
IV	10.1	67.9
V	13.9	64.5
VI	18.4	85.7
VII	19.4	76.7
VIII	20.7	76.2
IX	17.1	83.1

### Amendments

NaX-C were synthesized according to methods described by Panek et al.^54^, whereas NaX-Ver were synthesized with the addition of vermiculite (the Namekara mine, Mbale, Uganda). The selected properties of both zeolite composites are presented in Table 7. The specific surface area (S_BET_) of the NaX-C was about 1.3 times higher than NaX-Ver. More details about mineralogical composition and structural and textural characterization of applied zeolite composites are provided in the paper by Mokrzycki et al.^55^ Lignite was supplied by the Sieniawa Coal Mine (Poland).

**Table 7:** Selected properties of zeolite composites.

	pH (–)	EC (–)	K (mg g^−1^ d.m)	Mg (mg g^−1^ d.m)	Ca (mg g^−1^ d.m)	P (mg g^−1^ d.m)	Na (mg g^−1^ d.m)	Fe (mg g^−1^ d.m)	S (mg g^−1^ d.m)	Zn (mg g^−1^ d.m)	Σ PAHs (µg g^−1^ d.m)
NaX-C	10.30 ± 0.05	1.58 ± 0.02	7.05 ± 2.15	6.55 ± 1.60	8.30 ± 2.71	0.21 ± 0.07	23.96 ± 6.72	15.00 ± 2.31	1.05 ± 0.35	0.12 ± 0.04	36.11 ± 0.32
NaX-Ver	10.87 ± 0.09	2.01 ± 0.05	20.62 ± 3.90	27.70 ± 2.84	11.23 ± 1.50	0.18 ± 0.01	31.50 ± 2.24	7.03 ± 1.48	0.13 ± 0.01	0.02 ± 0.01	3.18 ± 0.02

### Extraction and analysis of PAHs

#### Determination of PAHs in plant and soil materials

Homogenized, air-dried soil samples (10 g) were mixed with DCM (100 mL) at room temperature and extracted using Soxtec with the following steps: (i) boiling in solvent for 90 min at 180 °C, (ii) rising step for 60 min, (iii) evaporation/solvent recovery for 15 min. Deuterated PAHs internal standard solutions (100 μL of phenanthrene-d10 at a concentration of 40 μg mL^−1^) were added to all samples. The solutions after Soxtec extraction were concentrated to a volume of 0.5 mL under nitrogen evaporation.

#### Plant materials

Ground roots, straw and grains (5 g) were mixed with 100 mL of solvent (HEX : DCM, 1:1, *v/v*) with the addition of anhydrous Na_2_SO_4_ (previously dried at 600 °C) and spiked with a labelled surrogated standard of phenanthrene-d10 (100 μL at a concentration of 40 μg mL^−1^)^56^. The extraction was performed using the Soxtec method (as described above). After conditioning the column with two portions of HEX (8 mL for each portion), samples were air-dried, solved with 4 mL of HEX and transferred into the column. The extract was eluted with two portions (8 mL) of a mixture of DCM and HEX (1:1, v/v). The extracts were purified on a column containing activated silica gel (10 g, Silica 100, Merck) topped with anhydrous Na_2_SO_4_ (5 g). The column was conditioned twice with 8 mL of hexane. The air-dried samples after Soxtec extraction were solved with 4 mL of HEX and transferred into the conditioned column. The samples of grains were additionally eluted with a two-portion (8 mL) of MeOH for fatty acids (FAs) analysis.

#### Determination of PAHs in plant and soil materials

The samples were concentrated to 0.5 mL under nitrogen evaporation. The content of 16 priority PAHs including naphthalene, acenaphthene, acenaphthylene, fluorene, phenanthrene, anthracene, fluoranthene, pyrene, benzo(a)anthracene, chrysene, benzo(b)fluoranthene, benzo(k)fluoranthene, benzo(a)pyrene, indeno(1,2,3-cd)pyrene, dibenzo(a,h)anthracene and benzo(g,h,i) perylene were determined using gas chromatography-mass spectrometry (GC/MS) (Agilent 7890A GC with 5975C MSD, Agilent Technology). The injection was done in on-column mode. The transfer line was set to 300 °C. The temperature program was: 99 °C for initial temperature, 2 °C min^−1^ to 310 °C for 34 min. Gas flow (He) 1.1 mL min ^−1^. The analysis was conducted with a 60 m column (DB-5MS, 250 μm, 0.25 μm), using QTM 16-PAHs mix (CRM47930, Sigma-Aldrich) as a standard. The LODs and LOQs of the 16 PAHs were 0.07–0.15 μg mL^−1^ and 0.20–0.45 μg mL^−1^, respectively. To ensure the accuracy and precision of this study's individual PAHS analysis process, a seven-point calibration standard (linearity R^2^ > 0.99; 0.5–12.5 μg mL^−1^) in solution, detection limits and procedural blank were carried out. Each calibration standard and sample contained an internal standard (100 μL of phenanthrene-d10 at a concentration of 40 μg mL^−1^). The recoveries ranged from 76 to 102% for individual PAHs. The reported results have been corrected for losses.

### Physicochemical characteristics of soils

The following analyses were carried out for soils: TOC, black carbon (BC), pH, EC, contents of N_total_ (TN) and heavy metals: Cd, Zn, and Pb. The content of TOC was measured according to Tiurin’s method^57^. The BC and TN analyses were conducted using CHNS-analyzer (Carlo Erba EA 1108). The soil materials were combusted briefly in a tube furnace at 375 °C for 18–24 h. Then, the samples were cooled, and about 25 mg of the materials were weighed and placed into silver tin capsules. To remove any remaining inorganic carbonates, two drops of 1 M HCl were added (HCl:water ratio of 1:1, v:v)^29^. The pH_H2O_ (distilled water), pH_KCl_ (1 M KCl)^58^ and EC (distilled water) were measured at a soil:solution ratio of 1:2.5 using ELMETRON CX-502 (Elmetron, Poznan, Poland).

### Dehydrogenase activity (DhA)

Dehydrogenase activity (DhA) was carried out according to the Thalmann method (1968)^59^, using triphenyltetrazolium chloride (TTC) as the electron acceptor. After incubation at 37 ± 2 °C for 24 h with TTC, samples were filtrated to separate the solution from soil and measured at 546 nm wavelength using a UV–VIS spectrophotometer (HITACHI U-5100, Hitachi High-Tech Science Corporation, Tokyo, Japan). The results are given as mean values from four analyses.

### Determination of Cd, Zn, and Pb concentration

The soil material (0.5 g) was mineralized using a mixture of 9 mL HNO_3_ and 3 mL HCl. The total concentration of Cd, Zn, and Pb were analyzed using inductively-coupled plasma optical emission spectrometry (Optima 7300DC Perkin Elmer, USA). Multi-elemental solutions of 1.000 mg dm^−3^ ICP Standard Certipur^®^ (Merck, Darmstadt, Germany) containing the analysed elements were used for calibration.

### Estimation of cancer risk

The BaP equivalent (BaP_eq_), based on the measured 4PAHs [benz(a)anthracene (BaA), benzo(b)fluoranthene (BbF), and chrysene (Chr)] concentrations and the corresponding toxic equivalence factors (TEFs), were calculated using the following equation:

1 $${BaP}_{eq}=\sum_{i=1}^{n}{C}_{i}\cdot {TEF}_{i},$$

where ${BaP}_{eq}$ is the total equivalent concentration of 4PAHs in maize grain (μg kg^−1^ d.m.); ${C}_{i}$ is the concentration of 4PAHs in the maize grain sample; and ${TEF}_{i},$ is the corresponding toxic equivalency factor (BaP = 1, Chr = 0.01, BaA = 0.1, BbF = 0.1)^60^.

### Data analysis

PAHs concentrations in all samples were calculated on the RBFs, SBFs and GBFs as follows:

2 $$RBFs=\frac{{C}_{roots}}{{C}_{soils}},$$

3 $$SBFs=\frac{{C}_{straws}}{{C}_{soils}},$$

4 $$GBFs=\frac{{C}_{grains}}{{C}_{soils}},$$

where ${C}_{soils}$, ${C}_{roots}$, ${C}_{straws}$, ${C}_{grains}$ represent the PAHs concentration in soils, roots, straws and grains, respectively.

### Statistical analysis

Statistical analyses were processed using Statistica version 13.0 software (StatSoft Inc., Poland). A one-way analysis of variance and Tukey’s post-hoc test (p > 0.05) were used to explore differences between the samples. Pearson’s correlation coefficients were also calculated. All of the figures were prepared using OriginPro2022 (OriginLab Corporation).

### Ethical statement

The study comply with relevant institutional, national, and international guidelines and legislation.

## Conclusions

The cultivation of maize on PAHs-degraded soils increases the risk of contaminating the pollutants in the edible parts of crops. The addition of zeolite composites mixed with lignite caused a marked decrease in Σ16 PAH levels in the soil and plant tissues (except for C9L6 in the grain) when compared to the control and NPK applied separately. It was evidenced that adding exogenous organic matter like lignite mixed with zeolite composites had a beneficial role in the degradation of PAHs in soils. In soils, the content of PAHs was correlated with pH_H2O_/pH_KCl_, EC, DhA, TN, and TOC for 5- and 6-rings PAHs. The lowest content of PAHs in straws was observed after the application of C3L3 and C9L6; however, in the case of grains, the best results were obtained for V3L3. Lower level of Σ16 PAHs in plant tissues was observed for NaX-C, which represented a higher specific surface area in comparison to NaX-Ver. Additionally, it could be concluded that the type of material used for zeolite-composites synthesis and the dose of applied additives influenced the accumulation of PAHs in particular parts of plants. Long-term stability tests and the impacts of applied zeolites on soils and soil micro-organisms should be evaluated in the future.

### Supplementary Information

Supplementary Table S1.

## Data Availability

The datasets generated and/or analysed during the current study are not publicly available but may be obtained from the corresponding author on reasonable request.

## Abbreviations

BaA: Benz(a)anthracene
B(a)P_eq_: Benzo(a)pyrene-equivalent
BbF: Benzo(b)fluoranthene
BaP: Benzo(a)pyrene
BC: Black carbon
C3L3: 3% NaX-C + 3% lignite + 94% NPK
C9L6: 9% NaX-C + 6% lignite + 85% NPK
V3L3: 3% NaX-Ver + 3% lignite + 94% NPK
V9L6: 9% NaX-Ver + 6% lignite + 85% NPK
Chr: Chrysene
DhA: Dehydrogenase activity
d.m.: Dry mass
HMW: High molecular weight
(K_ow_): Octanol–water partitioning coefficient
LMW: Low molecular weight
NaX-C: Zeolite–carbon composite
NaX-Ver: Zeolite–vermiculite composite
PAHs: Polycyclic aromatic hydrocarbons
Σ16 PAHs: The sum of 16 analysed PAHs
TOC: Total organic carbon
TN: Total nitrogen
UFA/SFA: Unsaturated fatty acids/saturated fatty acids
