# Supplementary material for: Restoration of soils contaminated with PAHs by the mixture of zeolite composites mixed with exogenous organic matter and mineral salts
Source: Sci Rep. 2023 Aug 30;13:14227. doi: 10.1038/s41598-023-41429-2 (PMC10469190; doi:10.1038/s41598-023-41429-2)
Supplement: Supplementary file 1 — Supplementary Table S1. [file 41598_2023_41429_MOESM1_ESM.docx]

Table S1. Concentration of individual PAHs in soils, roots, straws and grains of maize. Data represents means ± SD of four replications. Different letters in the columns indicate a significant difference (p<0.05) according to the HSD Tukey test for α = 0.05.

| Variant | Na | Acy | Ace | Flu | | Phe | An | Flur | Py | BaA | Chr | BbF | BkF | BaP | IcdP | dBAn | BPe |  |
| --- | --- | --- | --- | --- | --- | --- | --- | --- | --- | --- | --- | --- | --- | --- | --- | --- | --- | --- |
|  | Soil [mg kg^-1^] | | | | | | | | | | | | | | | | | |
| C | 0.101  ±0.010^a^ | 0.096  ±0.0010^a^ | 0.009  ±0.001^a^ | 0.032  ±0.002^a^ | | 0.154  ±0.016^a^ | 0.011  ±0.001^ab^ | 0.190  ±0.021^a^ | 0.144  ±0.021^a^ | 0.083  ±0.018^a^ | 0.111  ±0.010^a^ | 0.204  ±0.064^a^ | 0.059  ±0.009^ab^ | 0.095  ±0.024^a^ | 0.122  ±0.016^a^ | 0.024  ±0.001^a^ | 0.107  ±0.010^a^ |  |
| NPK | 0.067  ±0.016^b^ | 0.006 ±0.001^b^ | 0.005  ±0.00 ^b^ | 0.026  ±0.005^ab^ | | 0.130  ±0.031^abc^ | 0.007  ±0.001^ab^ | 0.122  ±0.011^bc^ | 0.093  ±0.009^b^ | 0.055  ±0.009^a^ | 0.064  ±0.003^c^ | 0.105  ±0.012^c^ | 0.038  ±0.003^c^ | 0.064  ±0.009^ab^ | 0.071  ±0.006^c^ | 0.014  ±0.002^c^ | 0.061  ±0.004^c^ |  |
| V3L3 | 0.063  ±0.011^bc^ | 0.004  ±0.003^bc^ | 0.005  ±0.001^b^ | 0.022  ±0.002^bc^ | | 0.110  ±0.015^abc^ | 0.007  ±0.001^ab^ | 0.114  ±0.019^bc^ | 0.085  ±0.013^b^ | 0.049  ±0.005^a^ | 0.060  ±0.008^c^ | 0.115  ±0.015^bc^ | 0.040  ±0.004^c^ | 0.062  ±0.005^b^ | 0.072  ±0.006^c^ | 0.013  ±0.002^c^ | 0.065  ±0.005^c^ |  |
| V9L6 | 0.072  ±0.012^b^ | 0.003  ±0.001^bc^ | 0.006  ±0.002^b^ | 0.021  ±0.003^bc^ | | 0.141  ±0.033^ab^ | 0.013  ±0.007^a^ | 0.169  ±0.059^ab^ | 0.131  ±0.046^ab^ | 0.078  ±0.033^a^ | 0.098  ±0.025^ab^ | 0.165  ±0.030^ab^ | 0.061  ±0.014^a^ | 0.085  ±0.021^ab^ | 0.098  ±0.014^b^ | 0.019  ±0.003^b^ | 0.088  ±0.016^ab^ |  |
| C3L3 | 0.036  ±0.010^c^ | 0.002  ±0.001^c^ | 0.004  ±0.001^b^ | 0.016  ±0.004^c^ | | 0.083  ±0.007^c^ | 0.005  ±0.0001^b^ | 0.102  ±0.007^c^ | 0.081  ±0.006^b^ | 0.050  ±0.006^a^ | 0.060  ±0.004^c^ | 0.097  ±0.011^c^ | 0.042  ±0.006^bc^ | 0.060  ±0.006^b^ | 0.075  ±0.008^bc^ | 0.013  ±0.001^c^ | 0.061  ±0.004^c^ |  |
| C9L6 | 0.059  ±0.013^bc^ | 0.004  ±0.002^bc^ | 0.005  ±0.002^b^ | 0.020  ±0.005^c^ | | 0.103  ±0.018^bc^ | 0.006  ±0.001^b^ | 0.129  ±0.013^abc^ | 0.107  ±0.011^ab^ | 0.063  ±0.006^a^ | 0.071  ±0.008^bc^ | 0.130  ±0.024^bc^ | 0.051  ±0.007^abc^ | 0.068  ±0.007^ab^ | 0.083  ±0.009^bc^ | 0.015  ±0.002^c^ | 0.076  ±0.009^bc^ |  |
|  | Roots [mg kg^-1^] | | | | | | | | | | | | | | | | |  |
| C | 0.049  ±0.003^c^ | 0.00059  ±0.00003^c^ | 0.0041  ±0.0002^d^ | 0.0191  ±0.0011^d^ | | 0.154  ±0.009^b^ | 0.0044  ±0.0002^e^ | 0.126  ±0.004^b^ | 0.571  ±0.032^a^ | 0.00185  ±0.00010^b^ | 0.0056  ±0.0003^b^ | 0.0052  ±0.0003^b^ | 0.0023  ±0.0001^b^ | 0.031  ±0.002^c^ | 0.0049  ±0.0003^b^ | - | 0.044  ±0.002^b^ |  |
| NPK | 0.092  ±0.005^a^ | 0.00088  ±0.00005^b^ | 0.0082  ±0.0004^a^ | 0.0329  ±0.0019^a^ | | 0.182  ±0.008^a^ | 0.0076  ±0.0005^a^ | 0.135  ±0.008^a^ | 0.584  ±0.021^a^ | 0.00426  ±0.00026^a^ | 0.0094  ±0.0006^a^ | 0.0094  ±0.0005^a^ | 0.0046  ±0.0002^a^ | 0.021  ±0.001^d^ | 0.0064  ±0.0004^a^ | - | 0.040  ±0.002^c^ |  |
| V3L3 | 0.088  ±0.003^a^ | 0.00133  ±0.00004^a^ | 0.0065  ±0.0002^b^ | 0.0291  ±0.0010^b^ | | 0.147  ±0.005^b^ | 0.0062  ±0.0002^c^ | 0.083  ±0.003^d^ | 0.373  ±0.012^d^ | 0.00073  ±0.00002^b^ | 0.0042  ±0.0001^c^ | 0.0037  ±0.0001^d^ | 0.0014  ±0.0001^d^ | 0.0127  ±0.0004^a^ | 0.0330  ±0.0011^c^ | - | 0.0063  ±0.0001^d^ |  |
| V9L6 | 0.075  ±0.002^b^ | 0.00126  ±0.00004^a^ | 0.0055  ±0.0002^c^ | 0.0263  ±0.0009^c^ | | 0.129  ±0.004^c^ | 0.0069  ±0.0002^b^ | 0.075  ±0.002^e^ | 0.377  ±0.012^d^ | 0.00099  ±0.00003^d^ | 0.0038  ±0.0001^d^ | 0.0037  ±0.0002^d^ | 0.0017  ±0.0001^c^ | 0.0127  ±0.0003^a^ | 0.0332  ±0.0011^c^ | - | 0.0052  ±0.0002^a^ |  |
| C3L3 | 0.044  ±0.001^c^ | 0.00060  ±0.00002^c^ | 0.0039  ±0.0001^c^ | 0.0161  ±0.0005^e^ | | 0.115  ±0.004^d^ | 0.0069  ±0.0002^b^ | 0.113  ±0.004^c^ | 0.563  ±0.018^b^ | 0.00134  ±0.00004^c^ | 0.0039  ±0.0001^d^ | 0.0042  ±0.0001^c^ | 0.0014  ±0.0001^d^ | 0.0128  ±0.0004^a^ | 0.0443  ±0.0015^a^ | - | 0.0086  ±0.0003^c^ |  |
| C9L6 | 0.020  ±0.001^d^ | 0.00049  ±0.00002^d^ | 0.0026  ±0.0001^f^ | 0.0133  ±0.0004^f^ | | 0.101  ±0.003^e^ | 0.0050  ±0.0002^d^ | 0.088  ±0.003^d^ | 0.415  ±0.013^c^ | 0.00138  ±0.00005^c^ | 0.0037  ±0.0001^d^ | 0.0036  ±0.0001^d^ | 0.0014  ±0.0001^d^ | 0.0117  ±0.0004^b^ | 0.0369  ±0.0012^b^ | - | 0.0066  ±0.0002^c^ |  |
|  | Straws [mg kg^-1^] | | | | | | | | | | | | | | | | |  |
| C | 0.030  ±0.001^b^ | 0.00277  ±0.00013^a^ | 0.0040  ±0.0002^a^ | 0.0137  ±0.0006^b^ | | 0.196  ±0.009^ab^ | 0.0138  ±0.0006^a^ | 0.470  ±0.022^a^ | 2.31  ±0.11^a^ | 0.0040  ±0.0002^a^ | 0.0165  ±0.0008^a^ | 0.0067  ±0.0003^a^ | 0.0133  ±0.0005^a^ | 0.031  ±0.001^a^ | 0.173  ±0.008^a^ | 0.055  ±0.003^c^ | - |  |
| NPK | 0.050  ±0.002^a^ | 0.00054  ±0.00002^b^ | 0.0034  ±0.0002^b^ | 0.0154  ±0.0007^a^ | | 0.203  ±0.009^a^ | 0.0126  ±0.0006^b^ | 0.414  ±0.019^b^ | 2.07  ±0.10^b^ | 0.0030  ±0.0001^c^ | 0.0141  ±0.0006^b^ | 0.0065  ±0.0003^a^ | 0.0108  ±0.0005^a^ | 0.026  ±0.001^b^ | 0.134  ±0.006^b^ | 0.077  ±0.004^a^ | - |  |
| V3L3 | 0.0197  ±0.0006^c^ | 0.000026  ±0.000008^c^ | 0.00244  ±0.00007^d^ | 0.0116  ±0.0004^c^ | | 0.197  ±0.006^b^ | 0.0128  ±0.0004^b^ | 0.382  ±0.013^c^ | 1.99  ±0.07^c^ | 0.0077  ±0.0003^c^ | 0.0077  ±0.0003^c^ | 0.0043  ±0.0001^c^ | 0.0086  ±0.0003^c^ | 0.021  ±0.001^c^ | 0.117  ±0.004^c^ | 0.075  ±0.002^c^ | - |  |
| V9L6 | 0.0226  ±0.0010^c^ | 0.00062  ±0.00002^c^ | 0.00165  ±0.00007^e^ | 0.0124  ±0.0006^e^ | | 0.181  ±0.008^c^ | 0.0113  ±0.0005^c^ | 0.346  ±0.016^d^ | 1.76  ±0.08^d^ | 0.0063  ±0.0003^c^ | 0.0068  ±0.0003^b^ | 0.0038  ±0.0002^b^ | 0.0103  ±0.0005^b^ | 0.031  ±0.001^a^ | 0.110  ±0.005^d^ | 0.046  ±0.002^b^ | - |  |
| C3L3 | 0.0373  ±0.0017^bc^ | 0.00038  ±0.00002^c^ | 0.00204  ±0.00009^c^ | 0.0099  ±0.0004^d^ | | 0.112  ±0.005^d^ | 0.0069  ±0.0005^d^ | 0.144  ±0.007^e^ | 0.65  ±0.03^e^ | 0.0049  ±0.0002^b^ | 0.0061  ±0.0002^d^ | 0.0029  ±0.0001^d^ | 0.0069  ±0.0003^d^ | 0.017  ±0.001^d^ | 0.052  ±0.002^e^ | 0.052  ±0.002^b^ | - |  |
| C9L6 | 0.0529  ±0.0024^b^ | 0.00043  ±0.00002^c^ | 0.00381  ±0.00017^c^ | 0.0093  ±0.0004^de^ | | 0.095  ±0.004^d^ | 0.0050  ±0.0002^d^ | 0.118  ±0.005^e^ | 0.56  ±0.03^e^ | 0.0052  ±0.0002^ab^ | 0.0046  ±0.0002^d^ | 0.0029  ±0.0001^e^ | 0.0054  ±0.0003^e^ | 0.014  ±0.001^e^ | 0.045  ±0.002^de^ | 0.029  ±0.001^d^ | - |  |
|  | Grains [mg kg^-1^] | | | | | | | | | | | | | | | | |  |
| NPK | 0.050  ±0.002^b^ | 0.00059  ±0.00002^b^ | 0.0019  ±0.0001^b^ | 0.0059  ±0.0002^a^ | 0.034  ±0.001^b^ | | 0.0017  ±0.0001^c^ | 0.017  ±0.001^b^ | 0.066  ±0.002^c^ | 0.00036  ±0.00001^b^ | 0.00141  ±0.00005^a^ | 0.00085  ±0.00003^a^ | 0.00055  ±0.00002^a^ | 0.00145  ±0.00005^a^ | 0.00092  ±0.00003^b^ | - | 0.0075  ±0.0002^c^ |  |
| V3L3 | 0.035  ±0.001^a^ | 0.00045  ±0.00001^a^ | 0.00160  ±0.00005^a^ | 0.0051  ±0.0002^a^ | 0.034  ±0.001^a^ | | 0.00177  ±0.00006^a^ | 0.0173  ±0.0005^a^ | 0.069  ±0.002^b^ | 0.00036  ±0.00001^a^ | 0.00120  ±0.00004^a^ | 0.00146  ±0.00005^b^ | 0.00093  ±0.00005^b^ | 0.0089  ±0.00003^b^ | 0.00072  ±0.00002^b^ | - | 0.0070  ±0.0002^b^ |  |
| V9L6 | 0.022  ±0.001^d^ | 0.00033  ±0.00001^c^ | 0.00162  ±0.00002^d^ | 0.0056  ±0.0002^a^ | 0.0246  ±0.0008^c^ | | 0.00145  ±0.00005^d^ | 0.0121  ±0.0009^b^ | 0.046  ±0.002^d^ | 0.00022  ±0.00001^b^ | 0.00064  ±0.00002^b^ | 0.00039  ±0.00001^e^ | 0.00030  ±0.00001^d^ | 0.00082  ±0.00003^c^ | 0.00066  ±0.00002^c^ | - | 0.0045  ±0.0001^d^ |  |
| C3L3 | 0.016  ±0.001^c^ | 0.00051  ±0.00002^d^ | 0.00129  ±0.00004^d^ | 0.0049  ±0.0002^b^ | 0.0289  ±0.0009^d^ | | 0.00139  ±0.00005^e^ | 0.0148  ±0.005^c^ | 0.059  ±0.002^e^ | 0.00029  ±0.00001^d^ | 0.00101  ±0.00003^d^ | 0.00063  ±0.00002^d^ | 0.00040  ±0.00001^c^ | 0.00086  ±0.00003^d^ | 0.00086  ±0.00003^d^ | - | 0.0065  ±0.0002^e^ |  |
| C9L6 | 0.044  ±0.001^c^ | 0.00078  ±0.00003^a^ | 0.00183  ±0.00005^c^ | 0.0057  ±0.0002^a^ | 0.034  ±0.001^b^ | | 0.00172  ±0.00006^b^ | 0.0205  ±0.0007^a^ | 0.085  ±0.003^a^ | 0.00034  ±0.00001^c^ | 0.000105  ±0.00003^c^ | 0.00084  ±0.00003^c^ | 0.00105  ±0.00003^a^ | 0.00136  ±0.00004^a^ | 0.00095  ±0.00003^a^ | - | 0.0089  ±0.0003^a^ |  |
